# Insurance Payor Status and Outcomes in Pediatric Sports-Related Injuries: A Rapid Review

**DOI:** 10.3390/clinpract15030052

**Published:** 2025-03-04

**Authors:** Katherine M. Kutzer, Lulla V. Kiwinda, Daniel Yang, John Kyle Mitchell, Emily J. Luo, Emily J. Harman, Stephanie Hendren, Kendall E. Bradley, Brian C. Lau

**Affiliations:** 1 Department of Orthopaedic Surgery, Duke University School of Medicine, Durham, NC 27710, USA; lulla.kiwinda@duke.edu (L.V.K.); daniel.yang@duke.edu (D.Y.); emily.luo@duke.edu (E.J.L.); 2 Department of Orthopaedic Surgery, University of North Carolina School of Medicine, Chapel Hill, NC 27599, USA; john_mitchell@med.unc.edu; 3 Department of Orthopaedic Surgery, Duke University Medical Center, Durham, NC 27710, USA; emily.j.harman@duke.edu (E.J.H.); kendall.bradley@duke.edu (K.E.B.); brian.lau@duke.edu (B.C.L.); 4 Medical Center Library & Archives, Duke University School of Medicine, Durham, NC 27710, USA; stephanie.hendren@duke.edu

**Keywords:** insurance, pediatric orthopedics, sports-related injuries, health disparities

## Abstract

**Introduction**: The rise in youth sports participation has led to an increase in pediatric sports-related injuries in the United States, contributing to growing healthcare costs and exacerbating socioeconomic disparities. Insurance payor status is a critical factor influencing access to care, treatment delays, and health outcomes. This study examines the association between insurance payor status and outcomes in pediatric sports-related injuries. **Methods**: A systematic review of the Medline database was conducted. Included studies reported insurance payor status and pediatric sports orthopedic patient outcomes following surgery. Outcomes included time to be seen by a provider, treatment access, complication and revision rates, postoperative Emergency Department (ED)/Urgent Care utilization, readmission rates, hospital length of stay, pain, functional scores, discharge destinations, return to activity, and follow-up. **Results**: A total of 35 studies comprising 535,891 pediatric patients were included. Publicly insured or uninsured patients consistently experienced significant delays in accessing care, with average wait times for clinic visits, imaging, and surgery up to six times longer compared to privately insured patients. These delays were associated with worsened injury severity, higher rates of postoperative complications, and poorer functional outcomes. Publicly insured patients were less likely to receive advanced treatments such as bracing or physical therapy, further compounding disparities. Minority groups faced delays even when controlling for insurance status. **Conclusions**: Public and uninsured pediatric patients face systemic barriers to timely and equitable care, resulting in worse outcomes following sports-related injuries. Future research should explore targeted solutions to ensure equitable care for this vulnerable population.

## 1. Introduction

Sports-related injuries within the United States (US) pediatric population have steadily increased in prevalence over recent years, aligning with the popularity of youth sports participation [1]. Trends in earlier sport specialization and shifts from seasonal to year-round play have also contributed to higher rates of injury [2,3]. Accordingly, demand for sports injury treatment also continues to rise, contributing to the healthcare cost burden on families and exacerbating disparities between them. Given the field’s limited understanding of how social factors impact this historically understudied population, developing a deeper understanding of the factors impacting recovery is crucial to ensuring equitable, high-quality care for pediatric patients aiming to return to play.

One of the factors consistently shown to impact healthcare outcomes is insurance payor status. Patients who are uninsured experience barriers to higher level care, lower operation frequency, and higher mortality rates. Moreover, differences in screening and increased severity at the time of evaluation in this population emphasize the salience of insurance status even prior to patient presentation [4,5,6,7]. Given its significant consequences pervasive throughout the trajectory of patient care, insurance status remains a pivotal factor warranting examination in the context of healthcare disparities.

This relationship between insurance status and healthcare outcomes is especially prevalent in the realm of orthopedics, with growing research examining how financial coverage mediates clinical decision-making and patient outcomes. Specifically, systematic reviews have elucidated associations between insurance status and outcomes in orthopedic trauma, spine, shoulder, hip, and knee surgeries [4,5,8,9,10]. However, we were unable to find similar reviews conducted within sports, and there was extremely limited data within pediatric sports, in particular. With regard to adult patients with sports-related injuries, there were a few studies available which demonstrated that public insurance or uninsured status is associated with limited access to orthopedic care and postoperative rehabilitation via physical therapy [11,12,13]. However, a systematic review examining the effect of insurance payor status on pediatric sports-related injury postoperative outcomes has yet to be performed.

Addressing this gap in orthopedic care is pivotal to ensure equitable treatment in the US pediatric population, especially with the increase in participation, competition, yearly duration of play, and early sport specialization. This study aims to comprehensively examine the effect of insurance payor status on outcomes in pediatric sports-related injuries. We hypothesize that public insurance status or uninsured status will be associated with worse postoperative outcomes compared to private insurance status in pediatric patients.

## 2. Methods

### 2.1. Literature Search and Screening

**Figure 1:**
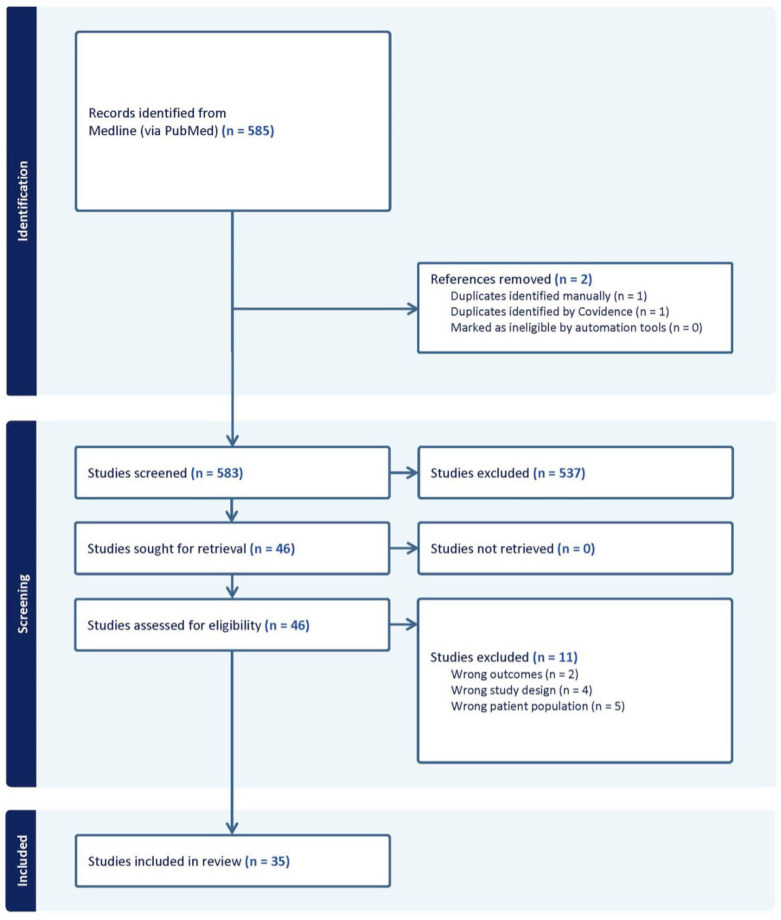
PRISMA flow diagram.

This systematic review followed PRISMA (Preferred Reporting Items for Systematic Reviews and Meta-Analyses) guidelines. The search protocol was not registered. Given the otherwise lack of comprehensive synthesis of evidence on this topic within the current literature, we conducted a rapid review, using only one database, instead of a full systematic review to meet the need for a timely overview of the research, while still adhering to the core principles of the systematic review process. A comprehensive search was conducted using Medline on 13 August 2024. A medical librarian performed a search using key terms designed to identify studies evaluating the relationship between pediatric orthopedic outcomes and insurance coverage. The search terms targeted insurance status, pediatric populations, and orthopedic injuries and treatments, as outlined in the detailed search strategies provided in Appendix A. The search yielded 585 records, which were imported into Covidence (Veritas Health Innovation, Melbourne, Australia), a systematic review management platform. Two duplicates were identified and removed. Inclusion and exclusion criteria were determined prior to data collection. Inclusion criteria involved original research studies in the United States analyzing pediatric patients (ages 0–18 years) who underwent orthopedic evaluation and/or surgery, which included insurance status relative to treatment access, patient outcomes, and/or healthcare utilization. Case reports, review articles, non-English texts, opinion pieces, letters to the editor, and studies with adult populations were excluded. After an initial screening of titles and abstracts by two independent reviewers, 46 articles were selected for full-text review. Of these, 35 studies met the inclusion criteria and were included in the final review (Figure 1). All voting disagreements at abstract and full-text levels were resolved with discussion. Manual data extraction was performed by one reviewer.

### 2.2. Quality Appraisal and Risk of Bias

All included studies were assessed for risk of bias and quality using the Methodological Index for Nonrandomized Studies (MINORS) criteria [14]. The MINORS criteria consist of a 12-item checklist, with each item scored as 0 (not reported), 1 (inadequately reported), or 2 (adequately reported). Maximum scores are 16 for non-comparative studies and 24 for comparative studies. The risk of bias and quality assessment is included in Table 1.

## 3. Results

### 3.1. Study and Cohort Characteristics

Across the 35 included studies ranging from 2005 to 2023, a total of 535,891 pediatric and adolescent orthopedic patients aged 0–18 years were analyzed to investigate the impact of insurance status on outcomes and access to care. The studies spanned diverse orthopedic conditions after sports-related injuries, including anterior cruciate ligament (ACL) injuries, meniscus tears [17,18,28,34,35,36,40,45,47,49], patellar instability [15,17,35,39], shoulder instability or dislocation [27,30], supracondylar humerus fractures [21,26,33,43], tibial spine fractures [44,46], and osteochondritis dissecans of the knee [17,37,46]. Insurance types included Medicaid, Medicare, private insurance, and uninsured patients. Most studies focused on delays in diagnosis and surgery, treatment course or follow-up, postoperative complications, and return to sport. Detailed study characteristics, including study design, sample size, and patient demographics are provided in Table 1.

### 3.2. Access to Care

**Figure 2:**
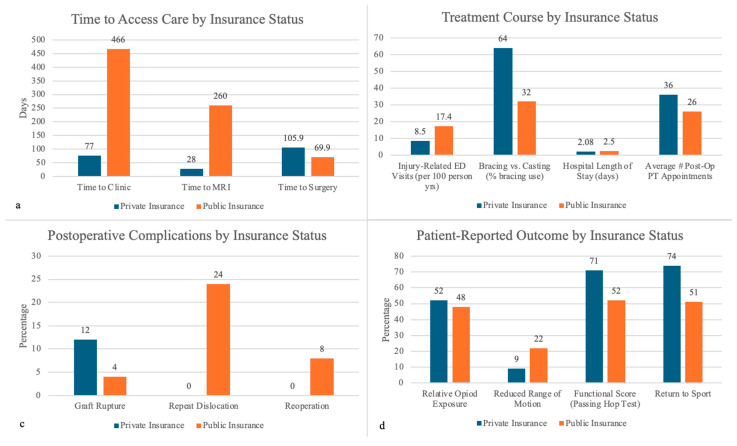
Summary of key findings. (**a**) Publicly insured patients experienced delays compared to privately insured patients, including longer times to clinic [15], imaging [35], and surgery [18]. (**b**) Treatment course shows publicly insured patients had more injury-related ED visits [42], higher rates of casting compared to bracing, longer hospital stays [37], and fewer postoperative physical therapy appointments [18]. (**c**) Postoperative complications revealed higher rates of repeat dislocation in publicly insured patients [27], while graft rupture was more common among privately insured patients [18]. Reoperation rates were higher in publicly insured patients [27]. (**d**) Patient-reported outcomes show that publicly insured patients experienced lower rates of functional recovery, including reduced range of motion [36], lower hop test pass rates [23], and lower return-to-sport clearance rates [18]. Privately insured patients were more likely to receive postoperative opioids [16].

Eighteen studies evaluated time to be seen and access to care, consistently demonstrating significant disparities based on insurance status (Table 2, Figure 2a). Publicly insured or uninsured patients faced substantial delays across all stages of care compared to privately insured patients. Allahabadi and colleagues reported that publicly insured patients waited, on average, 466 days from injury to their first clinic visit, compared to 77 days for privately insured patients (*p* = 0.002) [15]. Similar delays were noted for MRI scans, with publicly insured patients waiting 260 days compared to 28 days for private insurance holders [35]. These findings were corroborated by Beck and colleagues who showed that Medicaid patients faced delays in ordering and completing MRIs, with wait times more than doubling those of privately insured patients (*p* < 0.001) [17].

Delays extended beyond imaging to surgical intervention. Publicly insured patients waited 105.9 days on average for surgery, compared to 69.9 days for privately insured patients (*p* = 0.001) [18]. Similarly, Patel et al. demonstrated that Medicaid patients experienced nearly twice the delay to surgery (*p* < 0.0001) [36]. This was further supported by work regarding meniscal tears, supracondylar fractures, and shoulder instability [21,27,35].

Access to treatment was further hindered by systemic barriers. Medicaid patients were less likely to successfully schedule an appointment compared to those with private insurance. According to Hoch, 19.8% of patients with Medicaid compared to 1.0% of patients with Blue Cross Blue Shield (BCBS) were denied care simply based on their insurance coverage. Additionally, 13.5% of patients with Medicaid compared to 2.1% of patients with BCBS were denied appointments due to lack of a referral from a primary care physician [24]. Pierce et al. further expanded on these disparities, explaining that Medicaid reimbursement rates were often 23% the value of private insurance reimbursement [38].

Geographic variability also played a role. Interestingly, Medicaid patients in states that have not expended Medicaid [50] were twice as likely to secure appointments compared to those in expansion states, but still lagged behind privately insured patients in both regions [30]. Disparities were further exacerbated for minority groups; Bram et al. and Modest et al. found that Black and Hispanic children faced compounded delays, even when controlling for insurance type [18,33].

### 3.3. Treatment Course

**Figure 3:**
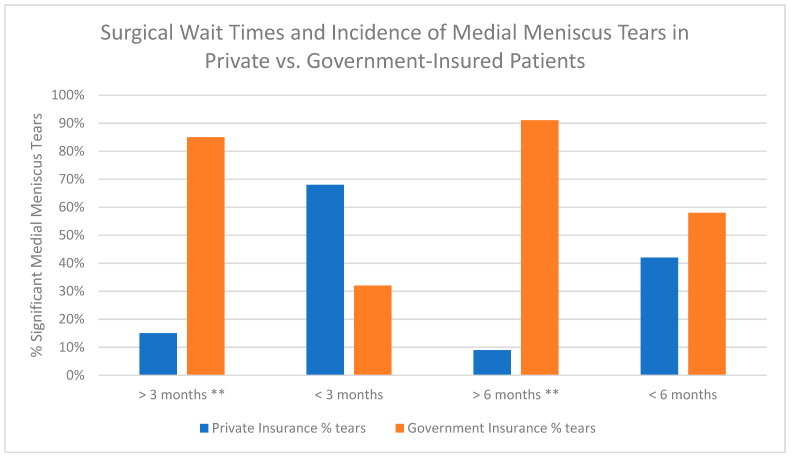
Surgical wait times and incidence of medial meniscus tears in private vs. government-insured patients. (Zoller 2017 [49])—This study shows a significant increase in medial meniscal tear incidence, decrease in preoperative scores, and worse tear severity with surgical wait time >6 months. Public insurance was a risk factor for longer surgical wait time and meniscus tear. There was a significant association between government insurance and surgical wait time >3 months (*p* < 0.001, OR 12.4), surgical wait time >6 months (*p* < 0.001, OR 7.8), and significant tears. ** *p* < 0.001.

**Figure 4:**
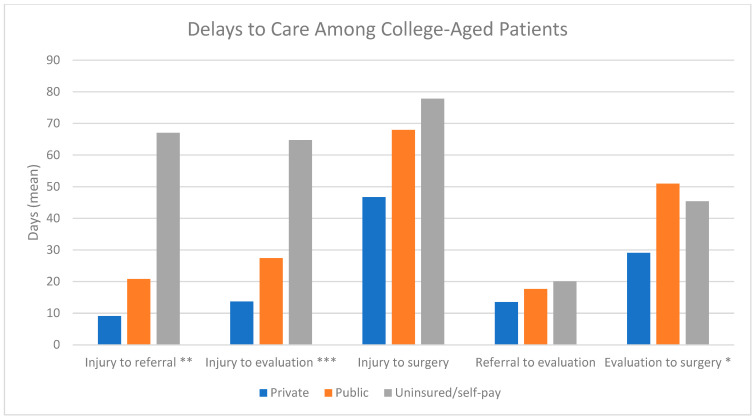
Delays to care among college-aged patients. (Johnson 2019 [28])—Publicly insured and uninsured pediatric and college-aged patients faced significant barriers in accessing orthopedic services, as demonstrated by substantially longer times between the initial injury and referral to an orthopedic evaluation and surgery; however, these socioeconomic factors did not affect the rate of surgical management. * *p* < 0.05, ** *p* < 0.001, *** *p* < 0.0001.

Twenty studies examined differences in treatment course based on insurance status, focusing on factors such as length of stay, discharge disposition, and follow-up (Table 3, Figure 2b). Publicly insured patients faced significant barriers to timely care, including delays in obtaining diagnostic imaging, surgery, and rehabilitation. Notably, Bram, Zoller, and Johnson demonstrated that longer surgical wait times for publicly insured patients were associated with more severe injuries, such as irreparable meniscal tears, further complicating treatment and recovery [18,28,49], Figure 3 and Figure 4. Access to rehabilitation was also disproportionately limited for publicly insured patients. Greenberg et al. showed that these patients had fewer weekly physical therapy visits and delayed post-surgical functional testing, leading to worse outcomes such as lower pass rates on hop testing (*p* = 0.0049) [23]. Moreover, the mean number of physical therapy visits for publicly insured patients was 26.7 compared to 36.4 for privately insured patients [18]. Sarkisova further discussed this disparity, finding that only 10.3% of rehabilitation centers accepted government-funded insurance, significantly restricting access for Medicaid patients [41].

Publicly insured patients were more likely to require emergency care. Simon et al. found that public insurance patients had significantly higher rates of injury-related visits (IRVs) to the Emergency Department (ED) (17.4 visits/100 person-years) compared to privately insured patients (8.5 visits/100 person-years) [42]. Li et al. also noted that while Medicaid patients visited the ED less frequently than privately insured patients (72.6% vs. 83.3%, OR = 0.640, *p* < 0.001) likely to avoid the cost, uninsured patients were significantly more likely to use emergency services (90.4%, OR = 1.881, *p* = 0.016) and faced higher charges [31]. These patterns reflect both delayed care and insufficient outpatient follow-up among patients with less comprehensive insurance coverage.

Systemic barriers also impacted discharge and follow-up care. Smith et al. found that publicly insured patients with tibial spine fractures were more likely to undergo postoperative casting rather than bracing, which is typically less favorable, reflecting limited access to advanced bracing options [46]. Gao et al. reported that publicly insured patients had a 20% longer hospital stay on average compared to privately insured patients, with higher daily charges despite limited treatment options [22].

### 3.4. Postoperative Complications

Six studies evaluated postoperative complications, examining disparities in secondary injuries, infections, emergency care visits, readmissions, and revision surgeries (Table 4, Figure 2c). Across these studies, publicly insured patients consistently faced worse outcomes compared to their privately insured counterparts, largely driven by delays in care and barriers to timely follow-up.

Publicly insured patients were more likely to experience secondary injuries and worse healing outcomes. Hung et al. reported that publicly insured patients with shoulder instability had a significantly higher rate of repeat dislocations compared to privately insured patients (24.3% vs. 0%, *p* = 0.022) [27]. Similarly, Patel et al. found that patients with osteochondritis dissecans (OCD) who had public insurance were far less likely to achieve union (20.5% vs. 79.5% for private insurance) [37]. These findings underscore how delays in care and limited resources exacerbate complications in public insurance groups.

Interestingly, publicly insured patients were less likely to undergo revision surgeries, even after experiencing complications [27]. This disparity is likely due to systemic barriers such as financial constraints and limited access to follow-up care, which prevent publicly insured patients from receiving corrective procedures. While Williams found no significant differences in overall surgical complication rates between private and public insurance groups (e.g., graft ruptures, infections, or arthrofibrosis, *p* = 0.36), the delayed presentation of publicly insured patients often resulted in more severe injuries requiring invasive procedures such as debridement instead of repair [47]. These findings emphasize how delays in care amplify the severity of complications.

### 3.5. Patient-Reported Outcomes

#### 3.5.1. Pain

Although none of the included studies focused on pain, Anandarajan et al. discussed opioid exposure. They found that patients with private insurance were not more likely to receive opioids but had a higher relative opioid exposure compared to those without private insurance, potentially reflecting disparities in pain treatment quality and access [16].

#### 3.5.2. Functional Scores

Functional recovery was significantly poorer among publicly insured patients. Greenberg et al. found these patients were 2.7 times less likely to pass functional hop tests for distance after ACL reconstruction due to reduced access to physical therapy during key rehabilitation phases [23]. Patel et al. observed that government-assisted patients experienced higher rates of decreased knee range of motion following ACL reconstruction compared to commercially insured patients (22% vs. 9%, *p* = 0.034) [36].

#### 3.5.3. Return to Sport

Publicly insured patients experienced significant delays in returning to activity. Greenberg et al. noted that these patients faced longer delays in completing functional tests and achieving readiness for sports participation [23]. Bram et al. reported lower sports clearance rates among publicly insured pediatric patients following ACL reconstruction, largely due to insufficient follow-up care and physical therapy access [18]. Rosenberg et al. linked socioeconomic disadvantages, such as lower neighborhood opportunity scores, to delayed surgical interventions, which in turn extended recovery times and impeded return to play [40].

These findings, as illustrated in Table 5 and Figure 2d, highlight systemic inequities in pain management, functional recovery, return-to-activity outcomes, and overall care accessibility for publicly insured pediatric patients, emphasizing the need for targeted interventions to reduce disparities.

### 3.6. Insurance Status and Other Social Drivers of Health

Although the primary focus of this work is on the relationship between insurance status and outcomes for pediatric orthopedic sports medicine patients, this work also highlighted the intersectionality of insurance status and other social drivers of health such as race/ethnicity [44]. For example, Bram et al. demonstrated significant delays to surgery and higher rates of irreparable meniscus tears resulting from Black/Hispanic race/ethnicity in addition to insurance status [15]. However, this study also found that Black/Hispanic patients were much less likely to be privately insured, with 54.3% of Black/Hispanic patients and only 16.0% of them having private insurance [15]. Similarly, although the work of Hung et al. supported the marked delays to care experienced by publicly insured pediatric and adolescent patients relative to those with private insurance, they go on to describe how additional factors such as the ability to attend appointments, including the ability to take time off work, have reliable transportation and access to child care, etc., as well as other factors such as access to postoperative physical therapy and distance from clinic may also play a role in explaining the delays to care seen in this patient population [35]. The interplay of numerous factors most likely contributes to the outcomes described in many of the studies we explored.

## 4. Discussion

This systematic review highlights significant disparities in pediatric sports related care based on insurance status, encompassing access to care, treatment course, postoperative complications, and patient-reported outcomes. The findings consistently demonstrate that patients with public or no insurance face worse outcomes compared to privately insured patients, driven by systemic barriers, delays in care, and limited access to rehabilitation and follow-up services. These disparities underscore critical gaps within the healthcare system that warrant targeted interventions for sports medicine providers to offer equitable and optimal orthopedic care for the pediatric population.

Race and ethnicity play a substantial role in shaping disparities in pediatric sports medicine care, compounding the inequities associated with insurance status. Multiple studies in this review demonstrated that minority patients, particularly Black and Hispanic children, face greater barriers to accessing timely and appropriate care. Modest et al. found that Hispanic and African American children were significantly more likely to receive inpatient treatment for fractures, suggesting potential disparities in treatment settings and access to outpatient services [33]. Similarly, Hubbard et al. reported that African American patients were three times more likely to miss follow-up appointments compared to their peers, indicating systemic challenges in care continuity [26]. Disparities also extend to surgical treatments, as Mercurio et al. highlighted that Black patients undergoing ACL reconstruction were more likely to receive meniscectomy rather than repair, a less favorable intervention associated with poorer long-term outcomes [32]. These findings underscore the complex interplay between race, ethnicity, and healthcare access, emphasizing the need for targeted strategies to address both structural barriers and implicit racial biases within the healthcare system.

Across the multiple social determinants of health discussed, the most striking was the role of transportation regarding access to care contributing to the six-fold increase in time from injury to first clinic visit for publicly insured patients compared to their privately insured counterparts [15]. Transportation barriers disproportionately affect families reliant on public insurance, limiting their ability to attend clinic appointments, imaging sessions, and rehabilitation services, which ultimately compounds injury severity and limits recovery outcomes [26,27]. Sarkisova et al. underscored the critical role of transportation support, highlighting how social work interventions can mitigate these challenges by arranging transportation for follow-up care, particularly for rehabilitation services, which are often inaccessible to Medicaid patients [41]. Addressing transportation barriers through solutions such as mobile clinics, telehealth, and subsidized transport programs could bridge these gaps and improve equitable access to pediatric sports medicine care.

### 4.1. Comparisons Within Orthopedics

These insurance-related disparities are consistent with findings from prior research in total joint arthroplasty, upper extremity procedures, and other orthopedic domains. In total hip and knee arthroplasty, Medicaid patients were shown to have significantly higher rates of postoperative complications, extended hospital stays, and higher overall costs following procedures such as total hip and knee arthroplasty [51,52]. Similarly, Medicaid patients undergoing shoulder arthroplasty exhibited higher rates of 90-day morbidity, readmissions, and reoperations [51].

Socioeconomic and systemic factors compound these disparities. Medicaid patients often face challenges such as limited access to high-volume surgical centers, which are associated with better outcomes [9]. They also had the highest rates of comorbidities among insurance groups and were more likely to be treated in inpatient care settings due to increased medical complexity, even for procedures that could otherwise be managed in outpatient settings [53]. These systemic issues highlight the intersection of insurance status, geographic location, and socioeconomic factors in shaping healthcare access and outcomes. While these factors are important in evaluating the interplay of social drivers of health in patient outcomes within this population, it was also important to understand the impact of insurance type independently from these additional challenges. It is therefore worth noting then, that where possible, a number of the studies evaluated conducted multivariate analyses to control for potentially confounding variables [15,23,25,33,35,40,41,44,48]. Following adjustment, these studies still found that even when controlling for factors such as age, race/ethnicity, location, language [33,40,44], hospital-level variabilities [23,35], and severity of injury [48], public insurance was still independently associated with negative outcomes. However, Slover et al., for example, still noted other variables such as education level that remain as potential confounding variables and were similarly not accounted for in other studies.

Despite these challenges, Medicaid patients have been shown to achieve comparable functional improvements to privately insured patients, but they start from a lower baseline, which contributes to worse absolute outcomes [52]. This finding underscores the need for tailored interventions that address baseline health disparities to improve overall outcomes in vulnerable populations.

### 4.2. Clinical and Policy Implications

The disparities identified in this review have important ethical implications, particularly in the context of health equity, justice, and policy. The consistent association between insurance status and worse clinical outcomes for pediatric patients with sports-related injuries raises important concerns about the equitable distribution of healthcare resources and access to timely, high-quality treatment. These findings support the idea that insurance type functions as a structural barrier that disproportionately affects certain patient populations. Addressing these disparities requires a multifaceted approach that extends beyond individual provider-level changes to systemic reforms aimed at reducing barriers to care. Several strategies identified through this work can be implemented to address these issues:

1. Streamlining Access to Diagnostic Services: Reducing administrative challenges, such as preauthorization requirements, and increasing provider networks that accept Medicaid could help mitigate delays in imaging and diagnosis [24,49].
2. Expanding Medicaid Reimbursement: Increasing reimbursement rates for Medicaid patients could incentivize more providers to accept publicly insured patients, improving access to both surgical and rehabilitation services [22,38].
3. Culturally Competent Care: Training healthcare providers to increase awareness of barriers to care (i.e., transportation), align their practice to support different communities (i.e., interpreters), and assist patients to overcome barriers (i.e., referral to charity care) could help reduce disparities in care access and outcomes [17,18].
4. Targeted Rehabilitation Support: Developing home-based or hybrid physical therapy programs for publicly insured and uninsured patients could help improve postoperative recovery for vulnerable populations [35,37].
5. Community-Based Interventions: Establishing school-based or community-based orthopedic clinics could enhance early detection and treatment for underserved populations, particularly in rural and urban Medicaid-dense areas [21,26].

While these suggested interventions may serve as a helpful starting point, future research should further investigate the mechanisms driving these disparities to develop more targeted interventions that promote equitable outcomes in pediatric sports medicine.

### 4.3. Strengths and Limitations

While this review provides a comprehensive analysis of disparities in pediatric orthopedic care, several limitations warrant consideration. First, this rapid review only included a single database. Most studies relied on retrospective designs, which present an inherent risk of biases such as recall bias and selection bias, thus limiting causal inferences. Additionally, variability in Medicaid policies and coverage across states complicates the generalizability of findings. Finally, with regard to generalizability, although choosing to focus exclusively on US-based studies allowed us to focus our work within the unique insurance structures and the associated social disparities that exist within the US healthcare system, we recognize that this limits the global applicability of these findings. These limitations present key opportunities for future research expanding on this initial review. However, the major strength of this review is the diversity of insurance payor groups in conjunction with a wide array of pediatric orthopedic procedures and outcomes analyzed.

## 5. Conclusions

This systematic review provides compelling evidence that insurance status, compounded by race and socioeconomic factors, significantly impacts access, treatment, and outcomes in pediatric sports medicine care. Publicly insured and uninsured patients consistently face longer delays, higher rates of secondary injuries, and worse functional outcomes. Targeted policy changes, including expanding Medicaid reimbursement and increasing access to diagnostic and rehabilitation services, are critical for reducing these disparities and ensuring equitable care for pediatric sports medicine patients.

## Figures and Tables

**Table 1:** Study and cohort characteristics.

Author, Year	Study Design (Retrospective, Prospective, etc.)	Number of Subjects	Age: Mean (SD), Median (SE/Range), or Range	Insurance Type (Medicaid, Private, Uninsured, etc.)	Insurance Type by Number: N (%)	Risk of Bias Assessment (MINORS Score)
Allahabadi 2022 [15]	Retrospective	78	15.3 (2.4)	Public, Private	Public: 38 (48.7%), Private: 40 (51.3%)	10
Anandarajan 2021 [16]	Retrospective	19,821	14.2 (3.1)	Non-Private, Private	Non-Private: 9462 (48.0%) Private: 10,359 (52.0%)	12
Beck 2020 [17]	Retrospective	168	14 (3)	Private, Government	Private: 70 (41.7%) Government: 98 (58.3%)	12
Bram 2020 [18]	Retrospective	915	15.0 (2.2)	Public, Private	Public: 164 (17.9%) Private: 751 (82.1%)	12
Brodeur 2022 [19]	Retrospective	20,170	3–19	Private, Federal, Workers’ compensation, Self-pay, Unknown	Private: 18,074 (89.6%) Federal: 1742 (8.6%) Workers’ compensation: 68 (0.3%) Self-pay: 263 (1.3%) Unknown: 23 (0.1%)	12
Dodwell 2014 [20]	Retrospective	25,315	3–20	Not covered, Private, Medicare, Medicaid, other	Not covered: 712 (2.8%) Private: 21,886 (86.5%) Medicare: 42 (0.2%) Medicaid: 1494 (5.9%) Other: 1181 (4.7%)	12
Fletcher 2016 [21]	Retrospective	2584	0–4 4–8 8–12 >12	Private, Public, Uninsured	Insurance status: (*n* = 2583) - Private: 1508 (58%) - Public: 919 (36%) - Uninsured: 156 (6%) Insurance status of * type 2 patients (*n* = 583) - Private: 313 (54%) - Public: 247 (42%) - Uninsured: 23 (4%) * these 583 patients are included in the total 2583	10
Gao 2010 [22]	Retrospective	3345	13.8	Public, Private	Public: 633 (18.9%) Private: 2712 (81.1%)	10
Greenberg 2022 [23]	Retrospective	281	15.7 (1.9)	Public, Private	Public: 128 (45.6%) Private: 153 (54.4%)	12
Hoch 2022 [24]	Prospective/Simulated survey	96 offices	Fictitious 16-year-old	Medicaid, BCBS	Number of calls: 192 - Medicaid: 96 - BCBS: 96	6
Hogue 2024 [25]	Retrospective	334,659 orthopedic sports medicine visits	NR	Public, Private	NR	12
Hubbard 2022 [26]	Retrospective	560	5.2	Government, Private, Uninsured	Government: 278 (63.5%) Private: 121 (27.6%) Uninsured: 39 (8.9%)	12
Hung 2020 [27]	Retrospective	55	14.81 (1.68)	Private, Public	Private: 18 Public: 37	12
Johnson 2019 [28]	Retrospective	332 traumatic meniscal tears, 237 included in the study	16.92 (2.72)	Public, Private, Uninsured	Public: 117 (49.4%) Private: 63 (26.6%) Uninsured: 57 (24.0%)	12
Kiani 2022 [29]	Retrospective	24,843	14.89 (10.39–19.37)	Private, Public, Uninsured, Other, Unknown	Pre-pandemic (Jan 2016 to Feb 2020) - Private: 10,345 (53.9%) - Public: 7261 (37.8%) - Uninsured: 166 (0.9%) - Other: 1237 (6.4%) - Unknown: 16,833 (87.7%) Intra-pandemic (March 2020 to June 2021) - Private: 3291 (58.3%) - Public: 1925 (34.1%) - Uninsured: 69 (1.2%) - Other: 348 (6.2%) - Unknown: 8 (0.1%) Comparing pre-pandemic to intra-pandemic *p* < 0.01 Odds Ratio (95% CI) of receiving an ACL reconstruction among pediatric patients from January 2016 to June 2021 - Private: reference - Public: 0.921 (0.857–0.989), *p* = 0.02 - Uninsured: 1.250 (0.926–1.686), *p* = 0.14 - Other: 0.950 (0.782–1.054), *p* = 0.21	12
Kirchner 2019 [30]	Prospective/Simulated survey	91 physician offices	Fictitious 16-year-old	Medicaid, BCBS	- Medicaid: 91 - BCBS: 91	6
Li 2021 [31]	Retrospective	2557	Mean age: - Nonoperative group: 15.3 - Operative group: 15.2 *p* = 0.662	Private, Medicaid, Self-pay, Other	Private: 1705 (66.7%) Medicaid: 609 (23.8%) Self-pay: 177 (6.9%) Other: 66 (2.6%)	12
Mercurio 2022 [32]	Retrospective	14,398	≤10: 438 (3%) 11–14: 4301 (30%) 15–18: 9659 (67%)	Private, Public, Other	Private: 7699 (53%) Public or other: (47%)	12
Modest 2022 [33]	Retrospective	Outpatient: 2484 Inpatient: 4595 Total: 7079	Outpatient: 5 (5.4, 2.3) Inpatient: 5 (5.3, 2.4)	Private, Federal, Self-pay	Outpatient: - Private: 2066 (83.2%) - Federal: 345 (13.9%) - Self-pay:73 (2.9%) Inpatient: - Private: 3862 (84.1%) - Federal: 611 (13.3%) - Self-pay: 122 (2.7%)	10
Newman 2014 [34]	Cohort	272	15.2 (2.12)	Private, Government-assisted, Uninsured	Private: 166 (61.48%) Government-assisted: 81 (30.00%) Uninsured: 23 (8.52%)	12
Olson 2021 [35]	Cohort	49	Public insurance:16.4 Private insurance:15.6	Public, Private	Public insurance: 32 (65.3%) Private insurance: 17 (34.7%)	12
Patel 2019 [36]	Retrospective review	127	15	Private, Government-assisted	Private: 68 (53.5%) Government-assisted: 59 (46.5%)	12
Patel 2021 [37]	Retrospective review	196 (204 lesions)	12.4 (2.8)	Private, Public	Private: 160 (81.6%) Public: 44 (18.4) * Number of lesions	12
Pierce 2012 [38]	Fictitious Patient	42	Contacted 42 orthopedic offices instead of patients.	Medicaid, Private	Offices accepting Medicaid: 6 (14.3%) Not accepting Medicaid: 36 (85.7%) Accepting private insurance: 42 (100%)	6
Poorman 2020 [39]	Retrospective	25,413	13.9 (2.5)	Private, Medicaid/Government, Unknown	Private: 13,824 (54.4%) Medicaid/Government: 9403 (37.0%) Unknown/Not reported: 2186 (8.6%)	12
Rosenberg 2023 [40]	Retrospective comparative study	415	- High or very high COI score: 15 (2.6) - Low or very low COI score: 17 (1.8) - *p* < 0.001	Public, Private, Other	High or very high COI score: - Public: 63 (34%) - Private: 117 (62%) - Other: 8 (4%) Low or very low COI score: - Public: 71 (162%) - Private: 22 (51%) - Other: 14 (6%)	12
Sarkisova 2019 [41]	Masked telephone interviews with PT facilities	54	Contacted 54 PT offices instead of patients.	Private, Government	The number of centers that accepted private insurance was significantly greater than the number that accepted government insurance (85.2% vs. 14.8%, *p* < 0.001).	6
Simon 2006 [42]	Stratified random-sample cross-sectional survey of EDs in the National Hospital Ambulatory Medical Care Survey	33,654	Age 0–2 SIRV: 0.7 (0.5–0.9) IRV: 17.2 (15.6–18.8) Age 3–5 SIRV: 1.8 (1.4–2.1) IRV: 11.8 (10.7–12.8) Age 6–12 SIRV: 3.8 (3.4–4.2) IRV: 8.7 (8.0–9.4) Age 13–18 SIRV: 5.1 (4.6–5.5) IRV: 12.4 (11.3–13.4)	Private, Public, Self-pay	NR	12
Slover 2005 [43]	Retrospective examination of Healthcare Cost and Utilization Project (HCUP) Kid’s Inpatient Database (KID).	5511	Humerus: 7.1 (1.8) Femur: 8.2 (2.3) Forearm: 10.9 (2.9)	Private, Medicaid, Self-pay, Other	Humerus - Private: 1863 (63.2%) - Medicaid: 762 (25.9%) - Self-pay: 223 (7.6%) - Other: 98 (3.3%) Femur - Private: 816 (63.3%) - Medicaid: 340 (26.4%) - Self-pay: 77 (6.0%) - Other: 57 (4.4%) Forearm - Private: 585 (70.9%) - Medicaid: 171 (20.7%) - Self-pay: 39 (4.7%) - Other: 30 (3.6%)	12
Smith 2022 [44]	Cohort study	368	11.7 (2.9)	Public, Private	Public: 141 (38.3%) Private: 227 (61.7%)	12
Smith 2021 [45]	Utilized the Pediatric Health Information System (PHIS) database	27,168	MAT: 16.6 (2.6) Repair/Meniscectomy: 15.4 (3.3)	Private, Public, Other/Unknown	Private: 13,602 (50.0%) Public: 12,202 (44.9%) Other/unknown: 1364 (5.0%)	12
Smith 2021 [46]	Retrospective cohort study	434	11.7 (3.0)	Public, Private	Public: 169 (38.9%) Private: 265 (61.1%)	12
Williams 2017 [47]	Retrospective study	119	15.0 (1.7)	Private, Public	Private: 49 (41.1%) Public: 70 (58.8%)	12
Xu 2022 [48]	Retrospective study	122	NR	Private (PPO, HMO), Public	Private: 80 (65.5%) - PPO: 67 (83.7%) - HMO: 13 (16.3%) Public: 42 (34.4%)	12
Zoller 2017 [49]	Retrospective study	121	16.1 (9–19)	Private, Government	NR	12

ACL—Anterior Cruciate Ligament; BCBS—Blue Cross Blue Shield; CI—Confidence Interval; COI—Childhood Opportunity Index; HMO—Health Maintenance Organization; HR—Hazard Ratio; IRV—Injury-Related Visit; MAT—Meniscal Allograft Transplantation; NR—Not Reported; OR—Odds Ratio; PPO—Preferred Provider Organization; SIRV—Sports Injury-Related Visit; *—subgroup of patients.

**Table 2:** Treatment access.

Author, Year	Insurance Type	Time to Be Seen	Treatment Access	Conclusion
Allahabadi 2022 [15]	Public, Private	Time from initial injury to clinic: - Public: 466 days - Private: 77 days *p* = 0.002 Time from initial injury to MRI: - Public: 466 days - Private: 82 days *p* = 0.003 Time from initial injury to surgery date: - Public: 695 days - Private: 153 days *p* = 0.0003 MRI scan before initial clinic visit: - Public: 44.7% - Private: 40.0% *p* = 0.85 Time from initial clinic visit to MRI scan: - Public: 25.4 days - Private: 12.6 days *p* = 0.23 Time from clinic visit to surgery: - Public: 226 days - Private: 73 days *p* = 0.002	NR	Significant delays were seen for pediatric and adolescent patients with patellar instability and public insurance (approximately 6 times longer to clinical evaluation, more than 5.5 times longer to obtain MRI, and 4.5 times longer to surgery) relative to injured patients with private insurance.
Beck 2020 [17]	Private, Government	Time from initial injury to 1st visit; days (range) - Private: 12 (3.5–92) - Government: 5 (1–41) *p* < 0.001 Time from 1st visit to MRI order; days (range) - Private: 0 (0–1) - Government: 24.5 (3.25–59) *p* < 0.001 Time from injury to MRI completion; days (range) - Private: 34 (16–124) - Government: 66.5 (38–136) *p* < 0.001 Time from 1st visit to MRI completion; days (range) - Private: 11 (4–24) - Government: 40 (23–74) *p* < 0.001 Time from MRI order to MRI completion; days (range) - Private: 9 (3–14) - Government: 16.5 (9–22) *p* < 0.001 Time from MRI completion to follow up; days (range) - Private: 6 (4–12) - Government: 17 (10–27) *p* < 0.001	NR	This study demonstrates that pediatric sports medicine patients with government insurance have significant delays in ordering, completion, and follow-up of knee MRI in comparison to those with private insurance plans, even though there is no significant difference in the rate of positive findings on imaging leading to operative treatment.
Bram 2020 [18]	Public, Private	Mean time to OR after injury (days) - Public: 105.9 ± 111.1 - Private: 69.9 ± 88.5 *p* = 0.001	NR	This study identified several disparities in the continuum of care for pediatric ACL injury. We found differences in delays to surgery, rates of irreparable meniscus tears, duration of postoperative follow-up, rates of athletic clearance, number of PT visits, postoperative strength and ROM, and graft rupture along the lines of race and insurance status. There were no differences in rates of contralateral ACL injury or new meniscus tear.
Fletcher 2016 [21]	Private, Public, Uninsured	NR	Odds Ratio (95% CI) of receiving any surgery, immediate surgery, postoperative follow-up, and delayed surgery for modified Gartland type II supracondylar humerus fractures (private insurance vs. government insurance/uninsured) - Immediate surgery: 1.14 (0.81–1.63), *p* = 0.45 - Delayed surgery: 2.46 (1.31–4.64), *p* = 0.01 - Any surgery (immediate and delayed): 1.65 (1.12–2.45), *p* = 0.01 - Seen for follow-up 2.39 (1.01–5.63), 0.04	Despite an equivalent number of privately insured and publicly insured patients undergoing immediate surgery for type II fractures, those with public or no insurance who were discharged were 2.46 times less likely to obtain outpatient surgery when compared to privately insured patients.
Hoch 2022 [24]	Medicaid, BCBS	Mean days to appointment in all states (*n* = 107) - Medicaid: 3.30 - BCBS: 3.43 *p* = 0.152 Mean days to appointment in expanded states (*n* = 52) - Medicaid: 3.70 - BCBS: 4.66 *p* = 0.145 Mean days to appointment in unexpanded states (*n* = 55) - Medicaid: 2.88 - BCBS: 2.38 *p* = 0.723	Successful appointments by state (expanded); *n* (%) - Kentucky - Medicaid: 9 (75.0) - BCBS: 10 (83.3) *p* = 0.586 - Louisiana - Medicaid: 1 (8.3) - BCBS: 7 (58.3) *p* = 0.007 - Iowa - Medicaid: 9 (75.0) - BCBS: 7 (58.3) *p* = 0.339 - Arizona - Medicaid: 4 (33.3) - BCBS: 5 (41.7) *p* = 0.586 Successful appointments by state (unexpanded); *n* (%) - North Carolina - Medicaid: 3 (25) - BCBS: 9 (75) *p* = 0.017 - Alabama - Medicaid: 5 (41.7) - BCBS: 6 (50.0) *p* = 0.723 - Wisconsin - Medicaid: 7 (58.3) - BCBS: 8 (66.7) *p* = 0.339 - Texas - Medicaid: 6 (50.0) - BCBS: 11 (91.7) *p* = 0.026 Barriers to care by insurance type and state expansion status; *n* (%) - ICID required - Medicaid: - Expanded: 9 (18.8) - Unexpanded: 7 (14.6) - BCBS - Expanded: 14 (29.2) - Unexpanded: 10 (20.8) *p* = 0.152 - Insurance status - Medicaid: - Expanded: 12 (25.0) - Unexpanded: 7 (14.6) - BCBS - Expanded: 0 (0.0) - Unexpanded: 1 (2.1) *p* < 0.001 - Referral required - Medicaid: - Expanded: 3 (6.3) - Unexpanded: 10 (20.8) - BCBS - Expanded: 1 (2.1) - Unexpanded: 1 (2.1) *p* = 0.007	For patients with first-time ankle sprains, access to care is more difficult using Medicaid insurance rather than private insurance, especially in Medicaid unexpanded states.
Hogue 2024 [25]	Public, Private	NR	Completed new visits for patients with public health insurance - Pre-pandemic in-person: 31.1% - In-pandemic telehealth visit: 30.6% *p* = 0.057	Telehealth is a viable method of care for a range of pediatric OSM conditions, providing a similar quality of care as in-person visits with a greater geographic reach. However, in its current format, reduced disparities were not observed in pediatric OSM THVs.
Hung 2020 [27]	Private, Public	Time to be seen (mean) - Public: 402.38 days - Private: 85.61 days *p* = 0.000009	NR	Public insurance status affected access to care and was correlated with the development of secondary bony injury and a higher rate of postoperative dislocations. Clinicians should practice with increased awareness of how public insurance status can significantly affect patient outcomes by delaying access to care—particularly if delays lead to increased patient morbidity and healthcare costs.
Johnson 2019 [28]	Public, Private, Uninsured	Injury to referral; days (SD) - Private: 9.11 (11.04) - Public: 20.83 (23.89) - Uninsured/self-pay: 67 (115.92) *p* < 0.001 Injury to evaluation; days (SD) - Private: 13.71 (10.34) - Public: 27.43 (27.01) - Uninsured/self-pay: 64.71 (98.62) *p* < 0.0001 Injury to surgery; days (SD) - Private: 46.72 (26.76) - Public: 67.97 (44.90) - Uninsured/self-pay: 77.85 (102.29) *p* = 0.05 Referral to evaluation; days (SD) - Private: 13.51 (15.30) - Public: 17.64 (19.13) - Uninsured/self-pay: 20.06 (27.45) *p* > 0.05 Evaluation to surgery; days (SD) - Private: 29.08 (21.87) - Public: 50.95 (40.14) - Uninsured/self-pay: 45.38 (127.00) *p* = 0.0029	NR	Publicly insured and uninsured pediatric and college-aged patients faced significant barriers in accessing orthopedic services, as demonstrated by substantially longer times between the initial injury and referral to an orthopedic evaluation and surgery; however, these socioeconomic factors did not affect the rate of surgical management.
Kirchner 2019 [30]	Medicaid, BCBS	Median days to appointment - All states (N = 35) - Medicaid: 3 - BCBS: 2 *p* = 0.01 - Expanded (N = 12) - Medicaid: 3 - BCBS: 2 *p* = 0.13 - Not expanded (N = 23) - Medicaid: 3 - BCBS: 2 *p* = 0.03	Appointment success by insurance type and state expansion status; number (%) - All states: - Medicaid: 36 (39.6%) - BCBS: 74 (81.3%) *p* < 0.001 - Expanded: - Medicaid: 13 (27.7%) - BCBS: 35 (74.5%) *p* < 0.001 - Unexpanded: - Medicaid: 23 (52.3%) - BCBS 39 (88.6%) *p* < 0.001 Barriers to care by insurance and expansion status; number (%) - PCP referral required - Medicaid - All states: 8 (8.8%) - Expanded: 1 (2.1%) - Unexpanded: 7 (15.9%) *p* = 0.07 - BCBS - All states: 0 - ED record required - Medicaid - All states: 9 (9.9%) - Expanded: 7 (14.9%) - Unexpanded: 2 (4.5%) *p* = 0.03 - BCBS - All states: 9 (9.9%) - Expanded: 6 (12.8%) - Unexpanded: 3 (6.8%) - ICID required - Medicaid - All states: 10 (11.0%) - Expanded: 7 (14.9%) - Unexpanded: 3 (6.8%) *p* = 0.09 - Private - All states: 7 (7.7%) - Expanded: 5 (10.6%) - Unexpanded: 2 (4.5%)	For a first-time shoulder dislocation, access to care is more difficult with Medicaid insurance compared with private insurance. Within Medicaid insurance, access to care is more difficult in Medicaid expanded states compared with unexpanded states. Medicaid patients in unexpanded states are twice as likely as those in expanded states to obtain an appointment.
Modest 2022 [33]	Private, Federal, Self-pay	The logistic regression showed Hispanic (OR: 2.386, *p* < 0.0001), Asian (OR: 2.159, *p* < 0.0001), and African American (OR: 2.095, *p* < 0.0001) patients to have increased odds of inpatient treatment relative to white patients. Injury diagnosis on a weekend had increased odds of inpatient management (OR: 1.863, *p* = 0.0002). Higher social deprivation was also associated with increased odds of inpatient treatment (OR: 1.004, *p* < 0.0001).	NR	There are disparities among race and socioeconomic status in the surgical setting of SCHF management. Physicians and facilities should be aware of these disparities to optimize patient experience and to allow for equal access to care.
Newman 2014 [34]	Private, Government-assisted, Uninsured	Time to surgery: - Multiple additional surgeries at time of ACL reconstruction: 3.3 months (median) - Single additional surgery at time of ACL reconstruction: 2.0 months (median) - No additional injuries: 1.6 months (median) Underwent ACL reconstruction significantly sooner: - If they were older at time of injury: (Hazard Ratio [HR], 1.2 per 1 year; 95% CI, 1.1–1.2; *p* < 0.0001) - Covered by private insurance plan: (HR, 2.0; 95% CI, 1.6–2.6; *p* < 0.0001) Median time to ACL surgery: - Private plan: 1.5 months (95% CI, 1.3–1.7) - Non-private plan: 3.0 months (95% CI, 2.3–3.3)	NR	The risk of delayed ACL surgery was significantly higher among pediatric and adolescent subjects who were less affluent, who were covered by a non-private insurance plan, and who were younger. This study also confirms previous studies that have reported an association between a delay in ACL surgery and the presence of additional knee injuries requiring operative treatment, accentuating the importance of timely care.
Olson 2021 [35]	Public, Private	Injury to surgery: mean (SD) in days - Public insurance: 347 (466) - Private insurance: 117 (179) - *p* < 0.01 - 95% CI of mean difference: 31 to 204 Injury to MRI: - Public insurance: 260 (260) - Private insurance: 28 (27) - *p* < 0.001 - 95% CI of mean difference: 31 to 201 Injury to clinic: - Public insurance: 212 (343) - Private insurance: 73 (168) - *p* < 0.01 - 95% CI of mean difference: 9.0 to 154 Clinic to surgery: - Public insurance: 136 (181) - Private insurance: 44 (40) - *p* < 0.01 - 95% CI of mean difference: 10 to 73 Clinic to MRI: - Public insurance: 36 (48) - Private insurance: 3.9 (5.9) - *p* < 0.001 - 95% CI of mean difference: 5.0 to 30 MRI to surgery: - Public insurance: 109 (177) - Private insurance: 36 (137) - *p* = 0.09 - 95% CI of mean difference: −2.0 to 43	NR	Publicly insured pediatric patients waited significantly longer for a diagnosis of meniscal tear compared with privately insured patients, even in a safety-net setting. These delays were not associated with greater tear severity or cartilage changes. Providers in all models of care should recognize that insurance status and the socioeconomic factors it represents prevent publicly insured patients from timely diagnostic points of care and strive to minimize the resulting delayed return to normal activity as well as the potential long-term clinical effects thereof.
Patel 2019 [36]	Private, Government-assisted	Injury to first appointment (days ± SD): - Private: 48.9 ± 57.1 - Government: 96.5 ± 85.4 - *p* = 0.003 Injury to MRI: - Private: 44.2 ± 83.3 - Government: 85.9 ± 80.8 - *p* = 0.021 Injury to surgery: - Private: 90.4 ± 83.7 - Government: 174.6 ± 122.2 - *p* < 0.0001 First appointment to surgery: - Private: 41.9 ± 65.2 - Government: 78.1 ± 71.8 - *p* = 0.0036	NR	Pediatric patients who have government-assisted plans may experience delays in receiving definitive injury management and be at risk for postoperative complications. Our findings suggest a significant discrepancy in time to treatment as well as rates of concomitant knee injuries and postoperative complications between government and private insurance types.
Sarkisova 2019 [41]	Private, Government	Time to be seen to their first PT appointment (days) - Private: 8.09 - Government: 8.67 - *p* = 0.33	Of the 54 PT centers that responded: - Accepted both insurance: 8 (10.3%) - Accepted private but reject government: 38 (70.3%) - Rejected MediCal/BCBS: 8 (10.3%) The number of centers that accepted private insurance was significantly greater than the number that accepted government insurance (85.2% vs. 14.8%, *p* < 0.001).	Our study found there was a significantly lower rate of children with government-funded insurance that had access to postsurgical rehabilitation.
Smith 2022 [44]	Public, Private	Timing from injury to surgery >21 days (*n* = 78) - Public: 39 (50.0%) - Private: 39 (50.0%) <21 days (*n* = 290) - Public: 102 (35.2%) - Private: 188 (64.8%)	NR	Patients who underwent delayed surgery for tibial spine fractures were found to have a higher rate of concomitant meniscal injury, longer procedure duration, and more postoperative arthrofibrosis when the surgery length was >2.5 h. Those who experienced delays in diagnosis or MRI, saw multiple clinicians, and had public insurance were more likely to have a delay to surgery.
Smith 2021 [46]	Public, Private	Patients who were seen surgically (*n* = 365) Public: 149 (40.8%) - Days b/w injury and MRI: Private: 216 (59.2%)		Children with public insurance and a tibial spine fracture were more likely to experience delays with MRI and surgical treatment than those with private insurance. However, there were no differences in the nature of the surgery or findings at surgery. Additionally, patients with public insurance were more likely to undergo postoperative casting rather than bracing.
Williams 2017 [47]	Private, Public	NR	There was a longer delay between the injury and initial clinic visit for patients with public insurance (56 6 83 days for private insurance; 136 6 254 days for public insurance; *p* = 0.02). The time elapsed between the initial clinic visit and surgery was not significantly different between the groups (35 6 26 days for private insurance; 35 6 35 days for public insurance; *p* = 0.81).	In adolescent patients with ACL or meniscal tears, patients with public insurance had a more delayed presentation than those with private insurance. They also tended to have more moderate-to-severe chondral injuries and meniscal tears, if present, that required debridement rather than repair. More rapid access to care might improve the prognosis of young patients with ACL and meniscal injuries with public insurance.
Zoller 2017 [49]	Private, Government	Surgical wait times (months) Private insurance (% of tears in each insurance group): >3 mo —15%. - *p* < 0.001, OR 12.4 <3 mo—68% >6 mo—9%. - *p* < 0.001, OR 7.8 <6 mo—42% Government insurance: >3 mo—85% <3 mo—32% >6 mo—91% <6 mo—58% There was a significant association between government insurance and surgical wait time >3 months, surgical wait time >6 months, and significant tears.	NR	This study shows a significant increase in medial meniscal tear incidence, decrease in preoperative scores, and worse tear severity with a surgical wait time >6 months. Public insurance was a risk factor for longer surgical wait time and meniscus tear.

ACL—Anterior Cruciate Ligament; BCBS—Blue Cross Blue Shield; CI—Confidence Interval; COI—Childhood Opportunity Index; ED—Emergency Department; HMO—Health Maintenance Organization; HR—Hazard Ratio; ICID—Insurance Identification Number; IRV—Injury-Related Visit; MAT—Meniscal Allograft Transplantation; MRI—Magnetic Resonance Imaging; NR—Not Reported; OR—Odds Ratio; OSM—Orthopedic and Sports Medicine; PCP—Primary Care Provider; PPO—Preferred Provider Organization; SIRV—Sports Injury-Related Visit.

**Table 3:** Injury and treatment course.

Author, Year	Insurance Type	Injury Type	Surgery	Concomitant Procedures	Follow-Up
Allahabadi 2022 [15]	Public, Private	Patellar instability	MPFL Reconstructive, MPFL Repair	No difference by insurance status in number of patients requiring concomitant procedures with MPFL surgery (68.4% vs. 62.5%; *p* = 0.58)	NR
Beck 2020 [17]	Private, Government	All charts had a “sports medicine diagnosis” of ligamentous/soft tissue injury, structural abnormality, instability, or inflammation. Excluded from the study were patients >18 years of age, a diagnosis other than sports medicine (i.e., tumor, infection, fracture), and/or a lack of health insurance. - Major: ACL tear, full thickness meniscus tear, osteochondritis dessicans (OCD), loose body/chondral fragment - Minor: Chondromalacia/synovitis, plica, discoid meniscus/partial meniscus tear, signs of prior patellar dislocation, hoffa pad edema	NR	NR	Time from initial injury to 1st visit; days (range) - Private: 12 (3.5–92) - Government: 5 (1–41) *p* < 0.001 Time from 1st visit to MRI order; days (range) - Private: 0 (0–1) - Government: 24.5 (3.25–59) *p* < 0.001 Time from injury to MRI completion; days (range) - Private: 34 (16–124) - Government: 66.5 (38–136) *p* < 0.001 Time from 1st visit to MRI completion; days (range) - Private: 11 (4–24) - Government: 40 (23–74) *p* < 0.001 Time from MRI order to MRI completion; days (range) - Private: 9 (3–14) - Government: 16.5 (9–22) *p* < 0.001 Time from MRI completion to follow up; days (range) - Private: 6 (4–12) - Government: 17 (10–27) *p* < 0.001
Bram 2020 [18]	Public, Private	ACL injury requiring reconstruction Concurrent meniscus tear - Public: 130 (79.3%) - Private: 530 (70.6%) *p* = 0.95	NR	Meniscectomy: - Public: 70/164 (42.7%) - Private: 214/751 (28.5%) *p* = 0.487	Follow-up time (days) - Public: 345.6 ± 299.3 - Private 479.4 ± 419.8 *p* = 0.01 Number of physical therapy visits -Public: 26.7 ± 13.3 - Private: 36.4 ± 16.9. *p* < 0.001
Fletcher 2016 [21]	Private, Public, Uninsured	Supracondylar humerus fractures (N = 2583) - Type 1: 1134 (43.9%) - Type 2: 583 (22.6%) - Type 3: 866 (33.5%) Type 2 fractures (N = 583) - Admitted for surgical fixation: 383 (65.7%) - Discharged from ED: 193 (34.3%) Patients discharged with type 2 fractures (N = 193) - Private: 72 (37.3%) - Public: 105 (54.4%) - Uninsured: 16 (0.83%)	Patients with Type 2 fractures discharged from ED (N = 193) - Surgical fixation: 59 (30.6%) - Closed reduction in clinic: 92 (69.4%)	NR	Odds ratio (95% CI) of receiving any surgery, immediate surgery, postoperative follow-up, and delayed surgery for modified Gartland type II supracondylar humerus fractures (private insurance vs. government insurance/uninsured) - Immediate surgery: 1.14 (0.81–1.63), *p* = 0.45 - Delayed surgery: 2.46 (1.31–4.64), *p* = 0.01 - Any surgery (immediate and delayed): 1.65 (1.12–2.45), *p* = 0.01 - Seen for follow-up 2.39 (1.01–5.63), 0.04
Gao 2010 [22]	Public, Private	- E886.0, tackles in sports that cause fall on same level from collision, pushing, or shoving, by or with other person - E917.0, striking against or struck accidentally by objects or persons in sports without subsequent fall - E917.5, striking against or struck accidentally by objects or persons in sports with subsequent fall.	NR	NR	Mean days of hospital stay - Public: 2.50 - Private: 2.08 *p* value not given
Greenberg 2022 [23]	Public, Private	ACL injury requiring reconstruction	Age at surgery; age (SD) - Private: 15.3 (2.0) - Public: 16.1 (1.7) *p* = 0.0003 Graft type (see below) *p* = 0.0432 Allograft - Private: 5 (3.3%) - Public: 14 (10.9%) BTB allograft - Private: 6 (3.9%) - Public: 13 (10.2%) HS auto (1 tendon) - Private: 27 (17.6%) - Public: 17 (13.3%) HS auto (2 tendon) - Private: 74 (48.4%) - Public: 53 (41.4%) HS auto (2 tendon) with allograft - Private: 4 (2.6%) - Public: 7 (5.5%) Quad autograft - Private: 25 (16.3%) - Public: 17 (13.3%) IT band autograft - Private: 10 (6.5%) - Public: 5 (3.9%) Other/Unknown - Private: 2 (1.3%) - Public: 2 (1.6%)	Patients with meniscus repairs, partial meniscectomies, and articular cartilage debridements were included. Patients with concomitant ligament injuries requiring repair/reconstruction (e.g., medial collateral ligament reconstruction) were excluded.	Time from surgery to first PT visit; months (SD) - Private: 0.34 (0.21) - Public: 0.38 (0.27) *p* = 0.1672 Time from surgery to hop test; months (SD) - Private: 7.7 (1.5) - Public: 8.3 (2.2) *p* = 0.0097 Average # of PT visits/week; # (SD) Total visits at time of hop test - Private: 1.04 (0.38) - Public: 0.92 (0.37) *p* = 0.0049 First 6 weeks - Private: 1.28 (0.48) - Public: 1.20 (0.55) *p* = 0.1815 Weeks 7–12 - Private: 1.26 (0.52) - Public: 1.06 (0.50) *p* = 0.0012 Weeks 13–24 - Private: 0.99 (0.45) - Public: 0.88 (0.45) *p* = 0.0408 >24 Weeks - Private: 0.77 (0.79) - Public: 0.75 (0.69) *p* = 0.8319
Hogue 2024 [25]	Public, Private	NR	NR	NR	Completed follow-up visits for patients with public health insurance - Pre-pandemic in-person: 35.0% - In-pandemic telehealth visit: 29.4% *p* < 0.001
Hubbard 2022 [26]	Government, Private, Uninsured	Supracondylar humerus fracture	NR	NR	Compliant with follow-up visits - Government: 323/351 (92%) - Private: 153/162 (94.4%) - Uninsured: 45/47 (95.7%) *p* = 0.5667
Hung 2020 [27]	Private, Public	Shoulder instability	All patients underwent arthroscopic surgical stabilization in the lateral decubitus position	Presence of secondary bone injuries - Public: 6/18 (33.3%) - Private: 25/37 (67.6%) *p* = 0.016 Incidence of anterior vs. other labral pathology - Public: 31/37 anterior (83.8%) - Private: 14/18 anterior (77.8%) *p* = 0.588	Injury to diagnostic MRI (mean) - Public: 431.97 days - Private: 99.11 days *p* = 0.0001 Injury to surgery (mean) - Public: 561.38 days - Private: 226.44 days *p* = 0.0066 There was no statistically significant difference between the 2 insurance cohorts for the time from clinic presentation to diagnostic MRI and time from MRI to surgery.
Johnson 2019 [28]	Public, Private, Uninsured	Meniscal tear	Total (*n* = 198) - Total or partial meniscectomy: 111 (56.1%) - Meniscal repair: 77 (38.9%) - Trephination: 2 (1.0%) - Debridement only: 8 (4.0%) Private insurance (*n* = 53) - Total or partial meniscectomy: 29 (54.7%) - Meniscal repair: 20 (37.7%) - Trephination: 0 (0%) - Debridement only: 4 (7.6%) Public insurance (*n* = 97) - Total or partial meniscectomy: 53 (54.6%) - Meniscal repair: 39 (40.2%) - Trephination: 2 (2.1%) - Debridement only: 3 (3.1%) Uninsured/self-pay (*n* = 48) - Total or partial meniscectomy: 29 (60.4%) - Meniscal repair: 18 (37.5%) - Trephination: 0 (0.0%) - Debridement only: 1 (2.1%)	NR	NR
Li 2021 [31]	Private, Medicaid, Self-pay, Other	Patellar instability	Percent of patients undergoing surgery - Private: 5.9% - Medicaid: 4.9% - Self-pay: 0.0% - Other: 4.5% *p* = 0.009	- 25 concomitantly performed chondroplasties - 17 lateral releases	NR
Mercurio 2022 [32]	Private, Public, Other	ACL injury requiring reconstruction	ACL reconstruction	Isolated ACLR (*n* = 6061) - Private: 3523 (58%) - Public/other: 2538 (42%) ACLR + Meniscal procedure (*n* = 8337) - Private: 4176 (50%) - Public/other: 4161 (50%) ACL reconstruction vs. ACL reconstruction with meniscal procedure - Primary insurance (public/other): 1.1 (1.02–1.20) *p* = 0.02 * Patients without private insurance had a 10% increase in the odds of concomitant meniscal procedures	NR
Newman 2014 [34]	Private, Government-assisted, Uninsured	ACL injury, concomitant meniscal and chondral injuries	ACL reconstruction	Categorized based on none, one, or multiple concomitant meniscal and chondral injuries	NR
Patel 2021 [37]	Private, Public	Osteochondritis dissecans in knee	Trans-articular drilling 76 (48.4%) Loose body removal, chondroplasty 28 (17.8%) Osteochondral autograft transfer 12 (7.6%) Fixation with bioabsorbable screw 10 (6.4%) Fixation with chondral darts 9 (5.7%) Osteochondral allograft transfer 9 (5.7%) Fixation with metal screw 6 (3.8%) Intercondylar notch drilling 4 (2.5%) Retro-articular drilling 3 (1.9%) * N = 157. Some patients underwent more than 1 of these procedures concurrently.	NR	Mean: 15.8 ± 6.4 months
Rosenberg 2023 [40]	Public, Private, Other	ACL injury, meniscus injury, chondral injury	ACL reconstruction	Meniscectomy no association between COI and meniscectomy (OR 1.6 [95% CI 0.9 to 2.8]; *p* = 0.12) or presence of a chondral injury (OR 1.7 [95% CI 0.7 to 3.9]; *p* = 0.20).	NR
Simon 2006 [42]	Private, public, self-pay	Sports injury- or non-sports Injury-related visits	NR	NR	ED visits per 100 person-years (95% confidence intervals) Private: - SIRV: 3.2 (1.7–4.8) - IRV: 8.5 (4.6–12.3) Public: - SIRV: 3.2 (1.3–5.1) - IRV: 17.4 (8.4–26.3) Self-pay: - SIRV: 3.0 (1.1–4.9) - IRV: (6.0–19.3) * SIRV = sports injury-related visit * IRV = injury-related visit
Slover 2005 [43]	Private, Medicaid, Self-pay, Other	Supracondylar humerus, femoral shaft, radius, and ulna forearm fracture	Humerus fractures - Closed reduction w/o Internal fixation - Closed reduction w/ internal fixation - ORIF femur fractures - Spica cast application - External fixator - Internal fixation w/or w/o closed reduction - ORIF Forearm fractures - Closed reduction w/o Internal fixation - Reduction w/ Internal fixation	NR	NR
Smith 2022 [44]	Public, Private	Tibial spine fractures	Arthroscopic fixation, open fixation, closed reduction	Meniscectomy	Mean follow-up (months): 10.0 ± 1.1
Smith 2021 [45]	Private, Public, Other/Unknown	Substantial meniscal deficiency	MAT - Private: 44 (65.5%) - Public: 17 (25.4%) - Other/unknown: 6 (9.0%) Repair/meniscectomy - Private: 13,558 (50.0%) - Public: 12,185 (45.0%) - Other/: 1358 (5.0%)	ACL reconstruction, osteochondral grafting or ACI, guided growth procedure	NR
Williams 2017 [47]	Private, public	ACL tear, meniscal tear	ACLR and meniscal repair	NR	The mean follow-up for this study was 5.6 months (range, 1–27 months).

ACI—Autologous Chondrocyte Implantation; ACL—Anterior Cruciate Ligament; ACLR—ACL Reconstruction; BCBS—Blue Cross Blue Shield; BTB—Bone-Patellar Tendon-Bone; CI—Confidence Interval; COI—Childhood Opportunity Index; HMO—Health Maintenance Organization; HR—Hazard Ratio; HS—Hamstring; MAT—Meniscal Allograft Transplantation; MPFL—Medial Patellofemoral Ligament; MRI—Magnetic Resonance Imaging; NR—Not Reported; OCD—Osteochondritis Dissecans; OR—Odds Ratio; ORIF—Open Reduction Internal Fixation; PCP—Primary Care Provider; PPO—Preferred Provider Organization; PT—Physical Therapy; *—Note.

**Table 4:** Post-treatment outcomes.

Author, Year	Insurance Type	Surgical Complications	Revision	ED/Urgent Care Visit	Conclusion
Hung 2020 [27]	Private, Public	NR	Incidence of repeat dislocation - Public: 9/37 (24.3%) - Private: 0/18 (0%) *p* = 0.022 Incidence of repeat operation - Public: 3/37 (8.1%) - Private: 0/18 (0%) *p* = 0.214 * Many publicly insured patients elected not to undergo revision surgery even when they had a repeat instability event	NR	Public insurance status affected access to care and was correlated with the development of secondary bony injury and a higher rate of postoperative dislocations. Clinicians should practice with increased awareness of how public insurance status can significantly affect patient outcomes by delaying access to care, particularly if delays lead to increased patient morbidity and healthcare costs.
Li 2021 [31]	Private, Medicaid, Self-pay, Other	NR	NR	Patients visiting the ED; percent (odds ratio, 95% CI), *p*-value - Private: 83.3% (reference), reference - Medicaid: 72.6% (0.640, 0.510–0.802), <0.001 - Self-pay: 90.4% (1.881, 1.123–3.151), 0.016 - Other: 93.9% (3.098, 1.118–8.583), 0.030	Patients with recurrent instability had higher odds of surgery, while Black and uninsured patients had lower odds of surgery. ED visits were associated with significantly higher charges compared to office visits, and Black patients had higher charges than white patients. Minority and uninsured patients may face barriers in access to orthopedic care.
Patel 2019 [36]	Private, Government-assisted	Decreased knee ROM (stiffness): - Government-assisted: 22% - Private: 9% x2 = 4.51, *p* = 0.034 Graft failure: - Government-assisted: 8% - Private: 6% x2 = 0.13, *p* = 0.72 Re-operation: - Government-assisted: 9% - Private: 10% x2 = 0.02, *p* = 0.88 Infection: - Government-assisted: 4% - Private: 0% x2 = 2.56, *p* = 0.11	NR	NR	Pediatric patients who have government-assisted plans may experience delays in receiving definitive injury management and be at risk for postoperative complications. Our findings suggest a significant discrepancy in time to treatment as well as rates of concomitant knee injuries and postoperative complications between government and private insurance types.
Patel 2021 [37]	Private, Public	Union: - Private: 140 (79.5%) - Public: 36 (20.5%) Nonunion: - Private: 20 (71.4%) - Public: 8 (28.6%)	NR	NR	In this study, Black children with OCD of the knee were significantly less likely to heal than were white patients, even when controlling for numerous other factors in a multivariate model. Although the exact etiology of this finding is unclear, future work should focus on the social, economic, and cultural factors that may lead to disparate outcomes.
Simon 2006 [42]	Private, Public, Self-pay	NR	NR	Visits per 100 person-years (95% confidence intervals) Private: - SIRV: 3.2 (1.7–4.8) - IRV: 8.5 (4.6–12.3) Public: - SIRV: 3.2 (1.3–5.1) - IRV: 17.4 (8.4–26.3) Self-pay: - SIRV: 3.0 (1.1–4.9) - IRV: (6.0–19.3) * SIRV = sports injury-related visit * IRV = injury-related visit	Sports and recreation are the leading causes of pediatric ED IRVs. Hispanic children, regardless of insurance status, had lower rates of SIRVs than white children, which helps explain the lower rate of nonfatal IRVs to EDs among Hispanic youth.
Williams 2017 [47]	Private, Public	During this time, 9 complications were noted: 5 graft ruptures 2 superficial infections treated with antibiotics 1 arthrofibrosis requiring arthroscopic lysis of adhesions 1 ultrasound documented superficial vein thrombosis (greater saphenous vein). All occurred in patients who underwent ACL reconstruction. Five were in patients with private insurance, and 4 were in patients with public insurance (*p* = 0.36).	NR	NR	In adolescent patients with ACL or meniscal tears, patients with public insurance had a more delayed presentation than those with private insurance. They also tended to have more moderate-to-severe chondral injuries and meniscal tears, if present, that required debridement rather than repair. More rapid access to care might improve the prognosis of young patients with ACL and meniscal injuries with public insurance.

ACL—Anterior Cruciate Ligament; BCBS—Blue Cross Blue Shield; CI—Confidence Interval; COI—Childhood Opportunity Index; HMO—Health Maintenance Organization; HR—Hazard Ratio; IRV—Injury-Related Visit; MAT—Meniscal Allograft Transplantation; MRI—Magnetic Resonance Imaging; NR—Not Reported; OR—Odds Ratio; PPO—Preferred Provider Organization; ROM—Range of Motion; SIRV—Sports Injury-Related Visit; * —Note.

**Table 5:** Patient reported outcomes.

Author, Year	Functional Score or Patient Reported Outcomes Score	Return to Sport or Other Outcomes	Conclusion
Beck 2020 [17]	NR	A systematic review of 11 studies comparing type and timing of ACL repair in a pediatric population showed that patients with delays in operative management were more than 30 times more likely to report instability postoperatively, while patients with early operative treatment were more likely to return to preinjury activity level.	This study demonstrates that pediatric sports medicine patients with government insurance have significant delays in ordering, completion, and follow-up of knee MRI in comparison to those with private insurance plans, despite the fact that there is no significant difference in the rate of positive findings on imaging leading to operative treatment.
Bram 2020 [18]	NR	Clearance for sports - Public: 83/164 (50.6%) - Private: 555/751 (73.9%) *p* < 0.001 Time to clearance for sports (days) - Public: 268.7 ± 64.2 - Private: 272.3 ± 71.7 *p* = 0.7	This study identified a number of disparities in the continuum of care for pediatric ACL injury. We found differences in delays to surgery, rates of irreparable meniscus tears, duration of postoperative follow-up, rates of athletic clearance, number of PT visits, postoperative strength and ROM, and graft rupture along the lines of race and insurance status. There were no differences in rates of contralateral ACL injury or new meniscus tear.
Gao 2010 [22]	NR	Mean hospital length of stay - Public: 2.5 days - Private: 2.08 days Mean charge per hospital day - Public: USD 7900 - Private: USD 8794 *p* value not given	The adjusted mean hospital length of stay was 20% higher for patients with a public payer (2.50 days) versus a private payer (2.08 days). The adjusted mean charge per day differed about 10% by payer type (public, USD 7900; private, USD 8794).
Greenberg 2022 [23]	Private vs. public odds ratio for passing the single hop test for distance - Unadjusted odds ratio 95% CI: 3.02 (1.48–6.13); *p* = 0.0024 - Adjusted odds ratio 95% CI: 2.72 (1.27–5.81); *p* = 0.0102 Private vs. public odds ratio for passing entire battery of single-legged hop tests - Unadjusted odds ratio 95% CI: 1.87 (1.12–3.12); *p* = 0.0161 - Adjusted odds ratio 95% CI: 1.74 (0.98–3.07); *p* = 0.0567	NR	Publicly insured patients average a lower number of weekly PT visits, experienced a longer delay from surgery to hop testing and were 2.7 times less likely to pass the single leg hop for distance test.
Hogue 2024 [25]	Relative to having private insurance: - Public insurance was associated with lower ratings related to provider’s efforts to include the patients in the treatment decision (*p* = 0.045) and provider’s efforts to explain the condition (*p* = 0.026). - Having public insurance was associated with lower ratings in all assessed questions (*p*< 0.05). - Public insurance was associated with lower ratings related to preparedness for video visits (*p* = 0.024), and quality of audio (*p* = 0.001), and video (*p* = 0.021) connections.	White patients, non-Hispanic patients, and patients with private insurance consistently had a higher proportion of maximum ratings for overall care, ease of scheduling, and the care provider’s concern and effort.	Telehealth is a viable method of care for a range of pediatric OSM conditions, providing a similar quality of care as in-person visits with a greater geographic reach. However, in its current format, reduced disparities were not observed in pediatric OSM THVs.
Patel 2019 [36]	Range of Motion Stiffness - Private: 9% (6/68) - Public: 22% (13/58)	Injury to return to play: - Private: 336.2 ± 130.4 - Government: 394.7 ± 153.6 - *p* = 0.044 Surgery to return to play: - Private: 255.7 ± 116.8 - Government: 238.9 ± 98.5 - *p* = 0.445	Pediatric patients who have government-assisted plans may experience delays in receiving definitive injury management and be at risk for postoperative complications. Our findings suggest a significant discrepancy in time to treatment as well as rates of concomitant knee injuries and postoperative complications between government and private insurance types.
Poorman 2020 [39]	NR	Patients identifying as white and female were more commonly admitted for patellar instability between 2007 and 2017. Males admitted for patellar instability were also significantly older than females.	Based on a PHIS database search, pediatric hospital admissions for patellar instability are steadily increasing. The majority of patients admitted for patellar instability are female, white, and have insurance other than Medicaid. Males admitted for patellar instability tended to be older than females admitted for the same.
Rosenberg 2023 [40]	NR	Patients with high or very high COI scores experienced a shorter time between injury and surgery compared with patients with low or very low COI scores (median (IQR) 53 days (53) versus 97 days (104); *p* < 0.001). After adjusting for insurance and race/ethnicity, patients with low or very low COI scores were more likely to undergo ACLR more than 60 days after injury (OR 2.1 [95% CI 1.1 to 4.0]; *p* = 0.02) (Table 2) and 90 days after injury (OR 1.8 [95% CI 1.1 to 3.4]; *p* = 0.04) (Table 3) compared with patients with high or very high COI scores. After controlling for insurance, BMI, and time to surgery, patients with low and very low COI scores were more likely to have a concomitant meniscus tear at the time of ACLR (OR 1.6 [95% CI 1.1 to 2.5]; *p* = 0.04) (Table 4) compared with patients with high and very high COI scores. After controlling for insurance and time to surgery, there was no association between COI and meniscectomy (OR 1.6 [95% CI 0.9 to 2.8]; *p* = 0.12). Similarly, there was no association between COI and chondral injury when adjusting for insurance, BMI, age, and race/ethnicity (OR 1.7 [95% CI 0.7 to 3.9]; *p* = 0.20).	As the COI score is independently associated with a delay between ACL injury and surgery as well as the incidence of meniscus tears at the time of surgery, this score can be useful in identifying patients and communities at risk for disparate care after ACL injury.
Sarkisova 2019 [41]	NR	Average delay to appointment scheduled (business days) ACL injury: Private—9.42; Government—7.05 Ankle injury: Private—7.58; Government—9.95 Back injury: Private—7.13; Government—9.06	Our study found there was a significantly lower rate of children with government-funded insurance that had access to postsurgical rehabilitation.
Simon 2006 [42]	NR	Hispanic race/ethnicity was associated with lower rates of SIRVs across all insurance types. After controlling for demographic factors and insurance, Hispanic children were less likely to have an SIRV than white children (odds ratio, 0.7; 95% confidence interval, 0.6–0.9).	Sports and recreation are the leading causes of pediatric ED IRVs. Hispanic children, regardless of insurance status, had lower rates of SIRVs than white children, which helps explain the lower rate of nonfatal IRVs to EDs among Hispanic youth.
Slover 2005 [43]	NR	Supracondylar humerus fractures - Black and Hispanic patients were more likely to receive closed reduction with internal fixation (percutaneous pinning) than white patients (*p* = 0.02). White patients were 9.3% less likely than Black patients and 5.1% less likely than Hispanic patients to receive closed reduction and casting of supracondylar humerus fractures, but they were more likely to receive either closed reduction without internal fixation or ORIF. Femoral shaft fractures - Patients with private insurance were 2.7% and 3.4% more likely to be treated with an external fixation device for a femoral shaft fracture than patients in the Medicaid and self-pay groups, respectively. Similarly, patients in the all other payer group were 6.6% to 7.3% more likely to receive this treatment for a femoral shaft fracture than the Medicaid and self-pay groups (*p* = 0.015). Forearm fractures - For pediatric patients admitted to the hospital, no significant differences existed in the treatment method chosen for forearm fractures across race, primary payer, or income groups.	This study did demonstrate statistically significant differences in the treatment of pediatric supracondylar humerus across racial groups, with Black and Hispanic patients being more likely to receive percutaneous pinning of these injuries than white. Private insurance patients were also more likely to have femoral shaft fractures treated with an external fixator device than patients with Medicaid or self-pay as their primary payer.
Smith 2021 [45]	NR	Patients who underwent MAT also had 2.0 times higher odds of being women (95% CI, 1.2–3.3; *p* = 0.01) and 2.0 times higher odds of being privately insured (95% CI, 1.1–3.6; *p* = 0.02). MAT was performed most frequently in the northeast (4.9/1000 meniscal surgeries) and least often in the south (1.1/1000 meniscal surgeries) (*p* < 0.001).	In the United States, pediatric and adolescent patients who underwent MAT were older and more likely to be female and have private insurance than those undergoing meniscal repair or meniscectomy. MAT was only performed in 17 of 47 children’s hospitals that perform meniscal surgery.

ACL—Anterior Cruciate Ligament; ACLR—Anterior Cruciate Ligament Reconstruction; CI—Confidence Interval; COI—Childhood Opportunity Index; HR—Hazard Ratio; IRV—Injury-Related Visits; MAT—Meniscal Allograft Transplantation; NR—Not Reported; OR—Odds Ratio; PT—Physical Therapy; ROM—Range of Motion; SIRV—Sports Injury-Related Visits; THV—Telehealth Visit.

## Data Availability

No new data were created or analyzed in this study. Data sharing is not applicable to this article.

## Appendix A. Database Search Terms

Database (including vendor/platform): Medline (via PubMed).

**Set #**	**Search Strategy**	**Results**
1 Insurance keywords	“Insurance, Health”[Mesh] OR insurance[tiab] OR insurances[tiab] OR insured[tiab] OR Medicaid[tiab] OR medi-cal[tiab]	274,958
2 Pediatric keywords	“Adolescent”[Mesh] OR “Child”[Mesh] OR “Child, Preschool”[Mesh] OR “Hospitals, Pediatric”[Mesh] OR “Infant”[Mesh] OR “Infant, Newborn”[Mesh] OR “Neonatology”[Mesh] OR “Minors”[Mesh] OR “Pediatrics”[Mesh] OR “Pediatric Anesthesia”[Mesh] OR “Pediatric Emergency Medicine”[Mesh] OR “Perinatology”[Mesh] OR “Puberty”[Mesh] OR adolescent[tiab] OR adolescents[tiab] OR adolescence[tiab] OR baby[tiab] OR babies[tiab] OR boy[tiab] OR boys[tiab] OR boyhood[tiab] OR child[tiab] OR childhood[tiab] OR children[tiab] OR “emerging adult”[tiab] OR “emerging adults”[tiab] OR girl[tiab] OR girls[tiab] OR girlhood[tiab] OR infant[tiab] OR infants[tiab] OR infancy[tiab] OR juvenile[tiab] OR juveniles[tiab] OR kid[tiab] OR kids[tiab] OR minors[tiab] OR newborn[tiab] OR newborns[tiab] OR neonatal[tiab] OR neonate[tiab] OR neonates[tiab] OR neonatology[tiab] OR neonatologist[tiab] OR neonatologists[tiab] OR preterm[tiab] OR prematurity[tiab] OR preadolescent[tiab] OR preadolescents[tiab] OR preadolescence[tiab] OR puberty[tiab] OR pubescent[tiab] OR pubescence[tiab] OR prepubescent[tiab] OR prepubescence[tiab] OR pediatric[tiab] OR pediatrics[tiab] OR paediatric[tiab] OR paediatrics[tiab] OR PICU[tiab] OR Pediatrician[tiab] OR pediatricians[tiab] OR paediatrician[tiab] OR paediatricians[tiab] OR pediatric[tiab] OR pediatrics[tiab] OR paediatric[tiab] OR paediatrics[tiab] OR stepchild[tiab] OR stepchildren[tiab] OR schoolchild[tiab] OR schoolgirl[tiab] OR schoolgirls[tiab] OR schoolboy[tiab] OR schoolboys[tiab] OR “school age”[tiab] OR “school aged”[tiab] OR toddler[tiab] OR toddlers[tiab] OR teen[tiab] OR teens[tiab] OR teenager[tiab] OR teenagers[tiab] OR teenaged[tiab] OR teenage[tiab] OR youth[tiab] OR youths[tiab] OR youngster[tiab] OR youngsters[tiab] OR “young person”[tiab] OR “young persons”[tiab] OR “young people”[tiab]	5,011,769
3 Sport injuries	“Orthopedics”[Mesh] OR “Tibial Meniscus Injuries”[Mesh] OR “Humeral Fractures, Distal”[Mesh] OR “Anterior Cruciate Ligament Reconstruction”[Mesh] OR ((“Sports”[Mesh] OR “Youth Sports”[Mesh] OR sport[tiab] OR sports[tiab] OR athlete[tiab] OR athletes[tiab] OR athletics[tiab] OR athletic[tiab]) AND (injur*[tiab] OR hurt[tiab] OR hurting[tiab] OR accident[tiab] OR accidents[tiab])) OR ((“anterior cruciate ligament”[MeSH] OR “Anterior Cruciate Ligament Injuries”[Mesh] OR “Anterior Cruciate Ligament”[tiab] OR ACL[tiab] OR “Meniscus”[Mesh] OR “Menisci, Tibial”[Mesh] OR meniscus[tiab] OR menisci[tiab]) AND (“surgery”[Subheading] OR “surgery”[tiab] OR surgical[tiab] OR operative[tiab] OR operation[tiab] OR postoperative[tiab] OR post-operative[tiab] OR “general surgery”[MeSH Terms] OR repair[tiab] OR repaired[tiab] OR reconstruction[tiab] OR reconstructive[tiab] OR augment[tiab] OR augmentation[tiab] OR injury[tiab] OR re-injury[tiab] OR injuries[tiab])) OR “supracondylar fracture”[tiab] OR “supracondylar fractures”[tiab] OR ((humerus[tiab] OR humeral[tiab]) AND (fracture[tiab] OR fractures[tiab] OR broken[tiab])) OR orthopedic[tiab] OR orthopaedic[tiab] OR orthopedics[tiab] OR orthopaedics[tiab]	226,099
4	1 AND 2 AND 3	585
